# Body Mass Index Is Associated with Impaired Semen Characteristics and Reduced Levels of Anti-Müllerian Hormone across a Wide Weight Range

**DOI:** 10.1371/journal.pone.0130210

**Published:** 2015-06-12

**Authors:** Jorunn M. Andersen, Hilde Herning, Elin L. Aschim, Jøran Hjelmesæth, Tom Mala, Hans Ivar Hanevik, Mona Bungum, Trine B Haugen, Oliwia Witczak

**Affiliations:** 1 Faculty of Health Sciences, Oslo and Akershus University College of Applied Sciences, Oslo, Norway; 2 The Morbid Obesity Centre, Vestfold Hospital Trust, Tønsberg, Norway; 3 Department of Endocrinology, Morbid Obesity and Preventive Medicine, Institute of Clinical Medicine, University of Oslo, Oslo, Norway; 4 Department of Morbid Obesity and Bariatric Surgery, Oslo University Hospital, Oslo, Norway; 5 Fertilitetsklinikken Sør, Telemark Hospital, Porsgrunn, Norway; 6 Reproductive Medicine Centre, Skåne University Hospital, Malmö, Sweden; Nanjing Medical University, CHINA

## Abstract

There is still controversy as to how body mass index (BMI) affects male reproduction. We investigated how BMI is associated with semen quality and reproductive hormones in 166 men, including 38 severely obese men. Standard semen analysis and sperm DNA integrity analysis were performed, and blood samples were analysed for reproductive hormones. Adjusted for age and time of abstinence, BMI was negatively associated with sperm concentration (B = -0.088, P = 0.009), total sperm count (B = -0.223, P = 0.001), progressive sperm motility (B = -0.675, P = 0.007), normal sperm morphology (B = -0.078, P = 0.001), and percentage of vital spermatozoa (B = -0.006, P = 0.027). A negative relationship was observed between BMI and total testosterone (B = -0.378, P < 0.001), sex hormone binding globulin (B = -0.572, P < 0.001), inhibin B (B = -3.120, P < 0.001) and anti-Müllerian hormone (AMH) (B = -0.009, P < 0.001). Our findings suggest that high BMI is negatively associated with semen characteristics and serum levels of AMH.

## Introduction

Like worldwide, the body mass index (BMI) in the Norwegian population is increasing. Data from national public health surveys show that the proportion of overweight (BMI ≥ 25 kg/m^2^) and obese (BMI ≥ 30 kg/m^2^) adults is steadily rising, and the largest weight gain is seen in the male population [1, 2]

Overweight and obesity have a negative effect on female fertility [3]. In comparison, few studies address the consequence of high BMI on male reproductive health. Epidemiological studies have indicated an increased risk of couple infertility with high male BMI [4–6], and disturbance of the the hypothalamic–pituitary–gonadal axis has been proposed as a mechanism for impaired fertility in overweight and obese men [7]. Only few studies describe the relationship between BMI and semen parameters in a general population. Of these, some authors reported no association between BMI and semen parameters [8–10], whereas others described a negative association between BMI and sperm concentration [11, 12], total sperm count [12, 13], normal sperm morphology or sperm motility [12, 14]. Studies on the relationship between BMI and sperm characteristics in men recruited from fertility clinics have reported a negative association for sperm concentration [15–20], sperm motility [16–18, 20] and numbers of spermatozoa with normal morphology [18, 19, 21]. Few studies have investigated DNA damage in spermatozoa and the relationship to BMI is still unclear [16, 18, 22].

Negative associations between BMI and serum levels of testosterone and sex hormone binding globulin (SHBG) is well established [9, 11, 15, 18], while luteinizing hormone (LH) and follicle stimulating hormone (FSH) seems to be unaffected by high BMI [9, 11, 18, 23]. As testosterone is converted to oestradiol by aromatase in adipose tissue, an increase in oestradiol might be expected when fat mass accumulates. There is evidence for increased oestradiol levels in obese males [10, 11, 18], however, this is not found in all studies [9, 23].

Anti-Müllerian hormone (AMH) and inhibin B are produced by the Sertoli cells and are possible markers of spermatogenesis [24–27]. While high BMI has been associated with decreasing inhibin B serum levels [11, 28, 29], few studies, with conflicting results, have examined if there is an association between BMI and AMH [12, 30, 31].

Our aim was to increase the knowledge about the associations between BMI and male reproductive characteristics by including men from the general population and a large group of obese and severely obese men. We also wanted to explore the relationship between serum AMH and BMI.

## Methods

### Participants

The study was conducted at the Faculty of Health Sciences, Oslo and Akershus University College of Applied Sciences (HiOA), Oslo, in collaboration with Department of Morbid Obesity and Bariatric Surgery, Department of Medicine, Oslo University Hospital, Oslo, Morbid Obesity Center, Vestfold Hospital Trust, Tønsberg and the fertility clinic, Fertilitetsklinikken Sør, Telemark Hospital, Porsgrunn, all in Norway.

Male participants aged 18 years and older were recruited between 2008 and 2013. Overweight and obese men were recruited through advertising in local newspapers, by public notices, from commercial weight loss programmes (Grete Roede AS, Nesbru, Norway) and from two regional public obesity clinics. Two groups of normal weight men were recruited. The first group consisted of men from the general population recruited by advertisement. The majority of men recruited by advertisement were young adults. The second group was recruited from a fertility clinic and was added to achieve a wider age distribution in the normal weight group. This group included men from couples with diagnosed female factor infertility, aged 35 years and older with BMI 18.5–24.9 kg/m^2^. Semen quality was not an inclusion factor. Upon entry, participants underwent measurement of height (cm) using a wall mounted stadiometer, and weight (kg) using a digital scale (Soehnle Professional, Backnang, Germany). Waist circumference (cm) was measured by a tape and percentage body fat was obtained using hand-to-hand bioelectrical impedance analysis (BF306, OMRON Healthcare Ltd, Milton Keynes, UK). Men recruited from the Telemark Hospital and Vestfold Hospital Trust had their measurements done by trained staff in the respective hospital, while men recruited from Oslo University Hospital had their measurements done at HiOA. In addition, information about smoking status, medical treatment and history of cryptorchidism or previous cancer disease was recorded. Participants received 300 NOK (approximately 36 EUR) upon attendance.

### Ethics statement

The study was approved by the Regional Committee for Medical and Health Research Ethics, South East, Norway. All participants provided a written informed consent.

### Semen analysis

Subjects were asked to abstain from ejaculating for 2–7 days prior to sample collection, as recommended by World Health Organization (WHO) guidelines [32]. The specific length of abstinence time was recorded. Participants received a pre-weighed sample container and semen sample was obtained by masturbation on-site or at home, if more convenient. Participants bringing the sample from home were instructed to avoid cooling of the sample by carrying it close to the body during transportation and to deliver sample within 2 hours after collection. All semen samples were incubated at 37°C immediately upon arrival. The on-site samples were liquefied for 30 min before analysis. Otherwise the sample was regarded as liquefied upon arrival. Standard semen analysis was performed according to WHO guidelines [32]. The HiOA laboratory participates in the European Society of Human Reproduction and Embryology external quality assurance programme for semen analysis. Wet preparations for sperm motility analysis were recorded by a microscope mounted video camera if the sample reached the laboratory within two hours after collection. Recording was only performed for samples collected at HiOA as such equipment was not available in the collaborating hospitals. All videos were sent to Reproductive Medicine Centre, Skåne University Hospital, Malmö, Sweden, for motility analysis. Semen volume, sperm concentration, total sperm count, sperm morphology and sperm vitality were assessed at HiOA. Morphology smears were stained by the Papanicolaou method [32] and analysed according to the Tygerberg strict criteria [33]. Vitality smears were stained with Sperm VitalStain (Nidacon, Mölndal, Sweden). Semen samples from participants collected at Vestfold Hospital Trust and Telemark Hospital were prepared in their respective laboratories and sent to HiOA for analysis within one week. Briefly, stained smears for assessment of sperm vitality and unstained smears for evaluation of sperm morphology were made. Semen for sperm concentration analysis was diluted 1:5 in buffer containing fixative and stored at 4°C and 100 ul aliquots of semen were frozen at -80°C.

Sperm chromatin structure assay (SCSA) was performed at Reproductive Medicine Center, Skåne University Hospital, Malmö, Sweden and details of the method are described elsewhere [34–36]. Briefly, SCSA is used to distinguish between single stranded and double stranded DNA by the use of the metachromatic stain acridine orange (AO). AO intercalated in intact DNA (double-stranded) emits green fluorescence, while AO intercalated with degraded DNA (single-stranded) emits red fluorescence. The extent of DNA damage is expressed as DNA fragmentation index (DFI %), based on the ratio between the red and the total (red plus green) fluorescence intensity, and is calculated from the DFI frequency histogram obtained by the flow cytometer. DFI in spermatozoa was analysed with a FACScan flow cytometer (Becton Dickinson, San Jose, CA, USA), equipped with an air-cooled argon ion laser. A total of 10 000 events were accumulated for each measurement at a flow rate 200–300 cells/sec. Analysis of flow cytometric data was carried out using the SCSASoft software (SCSA Diagnostics, Brookings, SD, USA). All samples were run in the same batch by one technician and the intra-laboratory coefficient of variation (CV) for DFI analysis was 4.5%.

### Hormone analysis

Blood samples were collected before 10 a.m. and centrifuged 30 minutes after collection. Serum and EDTA-plasma were aliquoted, frozen at -80°C and stored until further analyses were performed. Due to the distance between Vestfold Hospital Trust, Telemark Hospital and HiOA, blood samples were prepared in the respective laboratories, frozen at -20°C and sent to the HiOA laboratory. Inhibin B was measured at Labmedicin Skåne Malmö, Sweden. The other hormones were analysed at Hormone Laboratory, Oslo University Hospital. Total testosterone was quantified using competitive radioimmunoassay (RIA). Analytic coefficient of variation (CV_A_) was 11% at 0.6 nmol/l and 6% at 13.0 nmol/l (Orion Diagnostica, Espoo, Finland). Sex hormone binding globulin (SHBG) was determined using non-competitive luminometric assay (ILMA), CV_A_ was 4% at 12 nmol/l, 3% at 56 nmol/l and 6% at 106 nmol/l (Siemens Healthcare Diagnostics, Tarrytown, NY, USA). Follicle stimulating hormone (FSH) and luteinizing hormone (LH) were measured using non-competitive immunofluorometric assay (IFMA), FSH had a CV_A_ of 2% at 3.6 IU/l, 14.0 IU/l and 70.0 IU/l. LH had a CV_A_ of 5% at 4.3 IU/l and 35.0 IU/l and 6% at 96.0 IU/l (DELFIA kit PerkinElmer Life Science, Wallac Oy, Turku, Finland). Oestradiol (E2) was quantified using competitive fluoroimmunoassay (FIA), CV_A_ was 8% at 0.05 nmol/l and 2% at 0.49 nmol/l (PerkinElmer Life Science, Wallac Oy, Turku, Finland). For analysis of anti-Müllerian hormone (AMH) and inhibin B, enzyme-coupled immunosorbent assay (ELISA) was used. AMH CV_A_ was 6% at 31 pmol/l and 4% at 98 pmol/l. Inhibin B had a CV_A_ at 7% at 19 ng/l and 260 ng/l (Beckman Coulter, Bera, CA, USA). AMH analysis was performed according to the procedure introduced in June 2013 [37]. Free androgen index was calculated (total testosterone/SHBG x100).

### Statistical analyses

Mann-Whitney U test was used to compare semen parameters between normal weight men and men with BMI ≥ 35 kg/m^2^. Chi-square test with Yates’ correction for continuity was used to compare the proportions of normal weight men and severely obese men with semen characteristics below the WHO lower reference limits [38]. The proportions of men with serum testosterone below the lower local reference value were evaluated by Fisher’s exact test. Relationships between BMI and hormone or semen parameters were addressed by multiple linear regression analyses. All variables used in the multivariable analyses were continuous. As there was a significant difference in age between the BMI groups, all data analysed in the regression models were adjusted for age. In the analyses of the data on semen quality, results were also adjusted for time of abstinence, as number of days varied among the participants. Residual statistics were investigated for the multiple linear regression models. Dependent variables were square root or log transformed if the residual distribution violated the model assumptions. Semen parameters from incomplete semen samples were excluded from the statistical analyses.

Possible differences in the reproductive characteristics between normal weight men recruited from the fertility clinic and normal weight men recruited by advertising were addressed by Mann-Whitney U test. As there was a significant difference in age between the subgroups, hormone levels and semen parameters were compared in a subset encompassing the range of the 25^th^ to the 75^th^ percentile for age of the whole normal weight group (29–40 years).

Statistical analyses were performed using IBM SPSS Statistics 20, and a P-value of 0.05 or lower was considered statistically significant. Particular attention should, however, be directed towards smaller P-values, i.e., those below 0.01, because a considerable number of P-values have been calculated.

## Results

### Study population

In total, 166 men were included in the study after excluding men with azoospermia, cryptorchidism or previous chemotherapy treatment. Characteristics of the population are described in Table 1. The mean age was 38 years (22–61). The majority of the participants were from Norway or Northern Europe (95%) and a small minority from southern Europe, Asia, Africa and South America (5%). When divided into BMI groups, 27% of the participants were normal weight (BMI 18.5–24.9 kg/m^2^), 31% were overweight (BMI 25–29.9 kg/m^2^), 19% were obese (BMI 30–34.9 kg/m^2^) and 23% were severely obese (BMI ≥ 35 kg/m^2^). The normal weight group comprised 37 men recruited from the fertility clinic and 33 men recruited by advertising. Normal weight participants recruited from the fertility clinic had shorter time of abstinence (2 vs. 4 days, P = 0.012) and lower ejaculate volume (median: 3.1 vs 4.1 ml, P = 0.028) than age-matched men with normal weight recruited by advertisement. However, no significant differences were found for total sperm count, sperm concentration, sperm morphology, sperm vitality or for reproductive hormones between the two normal weight subgroups.

**Table 1:** Characteristics of the participants according to BMI groups.

BMI (kg/m^2^)	18.5–24.9	25–29.9	30–34.9	≥ 35
n	45	52	31	38
**BMI (kg/m** ^**2**^ **)**				
Median	23.1	27.5	32.5	43.2
Range	18.8–24.9	25.0–29.8	30.0–34.7	35.8–62.7
**Age (years)**				
Median	36	37	39	43
Range	24–54	22–59	23–61	22–59
**Waist circumference (cm)**				
Median	83	99	113	141
Range	73–98	79–112	98–130	118–179
**Body fat (%)**				
Median	17	25	33	41
Range	5–27	18–35	27–38	33–52
**Abstinence time (days)**				
Median	3	3	3	4
Range	1–7	1–14	1–14	2–20
**Non-smokers (%)**	78	88	87	79

BMI, body mass index.

### BMI and semen parameters

When comparing the normal weight group and the severely obese group, the latter group had significantly lower total sperm count, progressive sperm motility, normal sperm morphology and lower percentage of vital spermatozoa (Table 2). The median percentage DFI, as measured by SCSA was significantly higher in the latter group.

**Table 2:** Characteristics of semen parameters according to BMI groups, comparison between group 1 and group 4, and associations between BMI and semen parameters by multiple linear regression.

BMI (kg/m^2^)	18.5–24.9	25–29.9	30–34.9	≥ 35	1 vs. 4	Multiple regression		
BMI group	1	2	3	4	P	B	P	95% CI for B
**Sperm cons. (x10** ^**6**^ **/ml)**								
n	39	47	31	33				
Median	53	60	54.9	41.5	0.314	-0.088^b^	**0.009**	-0.153,-0.023
Range	1.3–222	3.6–350	3.8–305	3.0–281				
**Total sperm count (x10** ^**6**^ **)**								
n	39	47	31	33				
Median	205	190	244	121	**0.042**	-0.223^b^	**0.001**	-0.355,-0.091
Range	7–1862	7–601	6–1290	20–1127				
**Progressive motility (%)**								
n	21	31	25	17				
Median	63	41	43	30	**0.001**	-0.675	**0.007**	-1.156,-0.194
Range	17–74	1–76	10–70	0–43				
**Normal morphology (%)**								
n	31	40	28	30				
Median	5	3	3	2	**<0.001**	-0.078	**0.001**	-0.124,-0.032
Range	0–12	1–10	0–7	0–7				
**Vital sperm (%)**								
n	34	43	30	34				
Median	90	87	88	83	**0.002**	-0.006^b^	**0.027**	-0.001,-0.011
Range	56–97	41–97	50–96	40–95				
**DFI (%)**								
n	23	26	28	30				
Median	14	16	15	19	**0.030**	0.002^a^	**0.382**	-0.003,0.007
Range	8–39	3–67	6–57	8–85				

BMI, body mass index; DFI, DNA fragmentation index; B, regression coefficient; CI, confidence interval.

P-values for differences between group 1 and group 4 were calculated by Mann-Whitney U test. Associations tested by multiple linear regression were adjusted for age and time of abstinence. All variables in the regression analyses were continuous.

^a^, log transformed data

^b^, square root transformed data

When adjusting for age and time of abstinence in the multiple linear regression model there was a statistically significant inverse relationship between BMI and sperm concentration, total sperm count, progressive sperm motility, normal sperm morphology and percentage of vital spermatozoa. No significant relationship between BMI and DFI was observed in the multiple regression model adjusted for age and time of abstinence. A larger proportion of severely obese men had normal sperm morphology and progressive sperm motility below the WHO lower reference limits [38] than normal weight men (Table 3). The proportions of men with sperm concentration and total sperm count below the WHO lower reference limits did not differ between severely obese and normal weight men.

**Table 3:** Proportions of normal weight and severely obese men with semen parameters below the WHO lower reference limits [38].

BMI (kg/m^2^)	18.5–24.9	≥ 35		
	n (%)	n (%)	χ^2^ (df)	P
Sperm concentration < 15 mill/ml	6/41 (15)	5/32 (16)	<0.01 (1)	1.000
Total sperm count < 39 mill/ejaculate	5/41 (12)	7/32 (22)	0.62 (1)	0.430
Normal morphology < 4%	13/40 (33)	25/31 (81)	14.40 (1)	<0.001
Progressive motility < 32%	4/21 (19)	10/17 (59)	4.79 (1)	0.029

χ^2^, Chi-square value; df, degrees of freedom.

n (%), number (percentage) of participants with sperm characteristics below WHO lower reference limit/ group total.

Associations were tested by Chi-square test with Yates’ correction for continuity.

### BMI and reproductive hormones

Adjusted for age, BMI correlated negatively with serum levels of total testosterone, SHBG, inhibin B and AMH, but no association was found between BMI and free androgen index (Table 4). Of the severely obese men 34% had serum levels below the lower reference limit for total testosterone (10 nmol/l), whereas all the normal weight men had testosterone levels above this value (P = 0.001). A positive association was found between BMI and oestradiol, while no associations with BMI was found for FSH or LH.

**Table 4:** Characteristics of reproductive hormones according to BMI group, and associations between BMI and serum hormone levels by multiple linear regression.

BMI (kg/m^2^)	18.5–24.9	25–29.9	30–34.9	≥ 35	Multiple regression		
n	45	52	31	38	B	P	95% CI for B
**Total T (nmol/l)**							
Median	20.7	16.0	14.2	11.1	-0.378	**< 0.001**	-0.469,-0.289
Range	12.3–35.2	8.5–34.5	4.0–25.1	5.1–17.4			
**FAI**							
Median	54.4	55.2	60.9	50.5	-0.161	0.330	-0488,0.165
Range	32.6–97.7	28.7–128.6	25.3–141.3	21.1–101.8			
**Oestradiol (nmol/l)**							
Median	0.13	0.14	0.14	0.16	0.001	**< 0.001**	0.001,0.002
Range	0.09–0.20	0.07–0.23	0.09–0.19	0.11–0.22			
**SHBG (nmol/l)**							
Median	38	29	23	26	-0.572	**< 0.001**	-0.770,-0.380
Range	22–68	14–63	7–51	11–36			
**FSH (IU/l)**							
Median	4.1	4.3	3.7	4.3	-0.001^a^	0.906	-0.003,0.005
Range	1.1–14.9	1.0–11.0	1.4–14.2	1.6–12.2			
**LH (IU/l)**							
Median	3.8	3.4	3.1	3.9	0.001^a^	0.760	-0.003,0.004
Range	1.4–7.5	1.5–9.2	0.9–10.3	1.1–8.2			
**Inhibin B (ng/l)**							
Median	202	183	170	123	-3.120	**< 0.001**	-4.249,-1.958
Range	55–405	71–377	58–298	52–213			
**AMH (pmol/l)**							
Median	54	42	34	31	-0.009 ^a^	**< 0.001**	-0.014,-0.005
Range	19–129	14–169	8–176	8–114			

T, testosterone; FAI, free androgen index; SHBG, sex hormone binding globulin; FSH, follicle stimulating hormone; LH, luteinizing hormone; AMH, anti-Müllerian hormone; B, regression coefficient; CI, confidence interval.

Associations tested by multiple linear regression were adjusted for age. All variables in the regression analyses were continuous variables.

^a^, log transformed data.

## Discussion

In this study, BMI was negatively associated with all semen characteristics explored, except for DFI. We observed statistically significant negative associations between BMI and sperm concentration, total sperm count, progressive sperm motility, sperm vitality and normal sperm morphology. Severely obese men were more likely to have normal sperm morphology and progressive sperm motility below the WHO lower reference limits than normal weight men. We also found a statistically significant negative association between BMI and serum levels of total testosterone, SHBG, inhibin B and AMH, while serum oestradiol levels were positively associated with BMI.

Although there are variations in semen quality within each BMI category, we found a significant decline in all standard semen quality markers with increasing BMI. In other studies suggesting a negative effect of BMI on semen quality, usually one or two semen parameters are reported to be negatively associated to BMI. This includes a study comprising Danish military conscripts where lower sperm concentration and sperm count were found at high or low BMI, but no relation of BMI to other semen parameters [11]. A large number of participants were included, however few participants had BMI in the obese range (≥ 30 kg/m^2^). Lower ejaculate volume with increasing BMI and a decline in sperm concentration with increasing waist circumference was reported in another study, comprising American couples planning to conceive [13]. A large group of obese men were included, but as semen analysis was performed with methods other than the WHO standard analysis, the results may not be comparable to our semen parameters. Both studies were performed on men from the general population, which is in contrast to the majority of studies on BMI and semen quality where participants recruited are men with impaired spermatogenesis or men from infertile couples. Hammiche and co-workers found a negative effect of high BMI on sperm concentration and motility [39], another group reported a negative correlation with sperm morphology only [23], while a third study found decreasing ejaculate volume with increasing BMI and lower sperm count in severely obese men [18]. Two recent meta-analyses have investigated the association between BMI and semen characteristics or sperm concentration and total sperm count. MacDonald and co-workers were not able to show any association [40], but the analysis was limited by a low number of included studies. Sermondades group included a larger number of studies and more than 13 000 men [41]. No association was found between BMI and sperm concentration or total sperm count when comparing means across BMI groups. They found, however, a significantly increased risk of azoospermia and oligozoospermia in overweight and obese men, indicating that high BMI may affect sperm production. Both studies comprised men from both normal population and fertility clinics.

Evaluation of sperm DNA damage by SCSA has been proposed as an additional prognostic tool to standard semen analysis [42]. A high degree of DNA damage, measured as DFI, is predictive of prolonged time to pregnancy and increased risk of infertility [43, 44]. Still, the relationship between BMI and sperm DNA damage is less explored than conventional semen parameters. Increased DNA damage has been shown in overweight [16] and obese men [16, 18, 22] when compared to normal weight men. However, other studies found no association between BMI and DFI [12, 45, 46], including a Swedish study comprising men from infertile couples [47]. Although we found a difference in DFI between the groups with BMI ≥ 35 kg/m^2^ and normal weight, no significant difference was found in the analysis when adjusted for age and time of abstinence.

Our results for total testosterone and SHBG are in agreement with the majority of studies of BMI in relation to reproductive hormones [23]. The increased serum insulin levels seen in obese men leads to down regulation of SHBG synthesis in the liver [48, 49] while excess unbound testosterone is converted to oestradiol by aromatase in the adipose tissue. This mechanism could explain why we observed no association between BMI and free androgen index. However, free androgen index does not reflect levels of free testosterone within the testicle. Our observations of no relationship between BMI and serum levels of LH and FSH is supported by a majority of studies of overweight and obese men [50]. As serum levels of FSH seem to be sustained in overweight and obese men, a decrease in semen quality is likely to be influenced by other factors.

Adipose tissue contains aromatase catalyzing the conversion of testosterone to oestradiol, which can explain increased oestradiol levels in obese males. In addition, decreased levels of SHBG may increase the fraction of unbound oestradiol. We observed a positive association between BMI and oestradiol and the highest levels were seen in men with BMI ≥ 35 kg/m^2^. This observation is in line with other studies investigating oestradiol in relation to BMI [10–12, 18], although elevated levels of oestradiol are not found in all studies of overweight and obese men [23, 29].

We found a significant negative correlation between BMI and serum level of AMH and inhibin B, suggesting a possible link between adiposity and Sertoli cell function. The association between BMI and inhibin B has been reported consistently by others [9–11, 18], while the impact of high BMI on serum AMH, also produced by Sertoli cells, is still uncertain. A twin study reported reduced AMH levels in obese compared to normal weight men [31], but this result was opposed by a study of men with normal or reduced sperm concentration and maldecended testes [51]. Two other studies reported contrasting results as well, but as they included seversly obese men only, the narrow BMI range may be the cause. Different methodology and lack of standardization are problematic when comparing these studies. Although AMH analysis in three of the studies [12, 31, 52] were performed with the same antibody as in our analysis, a methodological change was introduced in 2013 due to a complement interference phenomenon causing results lower than expected [37]. To our knowledge, this is the first study to use the improved AMH method and our results strengthen the evidence for a relationship between BMI and AMH.

The broad BMI range and the large group of obese and severely obese men is a strength of our study, as is also the large group of men recruited outside fertility clinics. Furthermore, measures of height and weight were performed by trained staff members to avoid bias due to self-reporting. All semen samples were analysed strictly according to WHO standards in one single and experienced laboratory. Limitations to the study include the relatively low number of participants. In a recent meta-analysis [41] on BMI and male reproduction the majority of studies comprised 200 participants or more. However, only one study [14] included men with BMI ≥ 40 kg/m^2^. Second, recruitment to the study may result in a selection towards subfertile males, who might have interest in joining a study concerning fertility. Nevertheless, this potential selection bias should be comparable in all BMI groups. Third, some of our normal weight participants were recruited at a fertility clinic. By using female infertility and not semen quality as inclusion criteria, we hypothesized that these men would resemble those recruited by advertisement. This was supported by our subanalysis. Despite significant differences in time of abstinence and ejaculate volume, neither sperm concentration, total sperm count, normal sperm morphology nor sperm vitality differed between the two subgroups of normal weight men. As abstinence time and ejaculate volume are associated, the difference might be explained by stronger motivation among men recruited from a fertility center to comply with semen sample recommendations. Furthermore, the reproductive characteristics in participants recruited from obesity centers may differ from men with similar BMI recruited by advertisement.

The effect of weight loss on semen quality is still largely unexplored. Only one study has addressed the benefits of weight loss on semen quality in a study where obese men participated in a weight loss programme focusing on a healthy diet and daily exercise. Weight loss was reported to improve total sperm count and morphology. Whether this improvement was due to a reduction in BMI or to the improved lifestyle is uncertain. Further studies on how life style change and weight reduction affect semen quality in obese men could provide information of clinical value for management of infertile men with high BMI.

In conclusion, BMI was negatively associated with sperm concentration, total sperm count, progressive sperm motility and sperm morphology. A reduced semen quality was most pronounced in men with BMI above 35 kg/m^2^. We identified a significant negative association between BMI and AMH, as also observed with inhibin B. Our results indicate that both sperm production and sperm maturation are affected by high BMI.
